# Prevalence and Clinical Characteristics of Sleeping Paralysis: A Systematic Review and Meta-Analysis

**DOI:** 10.7759/cureus.53212

**Published:** 2024-01-30

**Authors:** Mahmoud T Hefnawy, Basma E Amer, Samar A Amer, Khaled Moghib, Yehya Khlidj, Bahaa Elfakharany, Adel Mouffokes, Zainab J Alazzeh, Nishant P Soni, Muhannad Wael, Mohamed E Elsayed

**Affiliations:** 1 Faculty of Medicine, Zagazig University, Zagazig, EGY; 2 Medical Research Group of Egypt Branch, Negida Academy, Arlington, USA; 3 Faculty of Medicine, Banha University, Banha, EGY; 4 Family Medicine, Royal College of General Practice, London, GBR; 5 Faculty of Public Health and Community Medicine, Zagazig University, Zagazig, EGY; 6 Faculty of Medicine, Cairo University, Cairo, EGY; 7 Faculty of Medicine, University of Algiers Benyoucef Benkhedda, Algiers, DZA; 8 Faculty of Allied Medical Sciences, Pharos University, Alexandria, EGY; 9 Internal Medicine, Faculty of Medicine, University of Oran 1 Ahmed Ben Bella, Oran, DZA; 10 Faculty of Medicine, Jordanian University of Science and Technology, Ar-Ramtha, JOR; 11 Medicine, Gujarat Medical Education and Research Society Medical College and Hospital, Ahmedabad, IND; 12 Urology, Saint Joseph Hospital, Jerusalem, PSE; 13 Faculty of Medicine, An-Najah National University, Nablus, PSE; 14 Department of Psychiatry, School of Medicine and Health Sciences, Carl von Ossietzky University of Oldenburg, Oldenburg, DEU

**Keywords:** hallucinations, ptsd, prevalence, isp, isolated sleep paralysis, sp, sleeping paralysis

## Abstract

Sleep paralysis (SP) is a mixed state of consciousness and sleep, combining features of rapid eye movement (REM) sleep with those of wakefulness. The exact cause of SP is unknown, and its prevalence varies among the studies. We aim to identify SP's global prevalence, the affected population's characteristics, and the SP's clinical picture. We searched three databases (PubMed, Scopus, and Web of Science (WoS)) using a unique search strategy to identify eligible studies. All observational studies identifying the prevalence or frequency of sleeping paralysis were included. No exclusions are made based on country, race, or questionnaire. The analysis was performed using the latest version of R software (R Core Team, Vienna, Austria). The analysis included 76 studies from 25 countries with 167,133 participants. The global prevalence of SP was 30% (95% CI (22%, 39%)). There were similar frequencies of isolated SP and SP (33%, 95% CI (26%, 42%), I2 = 97%, P <0.01; 31%, 95% CI (21%, 43%), I2 = 100%, P = 0, respectively). A subgroup analysis showed that the majority of those who experienced SP were psychiatric patients (35%, 95% CI (20%, 55%), I2 = 96%, P <0.01). The prevalence among non-psychiatric patients was among students (34%, 95% CI (23%, 47%), I2 = 100%, P = 0). Auditory and visual hallucinations were reported in 24.25% of patients. Around 4% had only visual hallucinations. Meta-regression showed no association between the frequency of SP and sex. Publication bias was detected among the included studies through visual inspection of funnel plot asymmetry. Our findings revealed that 30% of the population suffered from SP, especially psychiatric patients and students. The majority of SP cases lacked associated hallucinations, while a noteworthy proportion experienced combined visual and auditory hallucinations.

## Introduction and background

Sleep paralysis (SP) is a mixed state of consciousness and unconsciousness, combining features of rapid eye movement (REM) with those of wakefulness [1]. During REM there is a generalized muscular atonia which is a natural pattern of normal REM sleep, possibly driven by gamma-aminobutyric acid (GABA) and glycine-mediated inhibition of motor neurons [2]. This state of skeletal muscle paralysis is associated with significant alveolar ventilation and tidal volume reductions, leading to hypercapnia during REM sleep [3]. In SP, patients become suddenly conscious of these physiological changes, which leads to acute night terrors, dyspnea, and suffocation sensations. Notably, patients can feel pressure on the chest, breathing difficulties, and pain, which are attributed to the effects of hyperpolarization of motoneurons on perceptions of respiration [4]. Furthermore, a hallucination-inducing process can occur, exacerbating the parasomnia experience, which is a state between sleep and wakefulness in which a person can perform abnormal behavior like talking or walking [4,5]. This process is believed to develop in the midbrain [4]. An electroencephalogram investigation of SP episodes revealed an intermediate spectrum between wakefulness and REM sleep in the alpha, theta, and delta frequency episodes, confirming a false awakening process during a dreaming state [5].

The reported risk factors include young age, increased BMI, smoking, alcohol consumption, poor sleep quality, anxiety disorders, and exposure to traumatic events [3,5,6]. Additionally, familial history was associated with higher odds of SP, hinting at the presence of genetically predisposed individuals to this condition [3,6]. Other risk factors include hypertension, idiopathic hypersomnia, insufficient sleep syndrome, narcolepsy, obstructive sleep apnea, and Wilson’s disease [7]. Recurrent SP can lead to impaired sleep quality and vice versa [1,8]. Moreover, it can lead to worse mental health-related quality of life among patients with sleep disorders such as obstructive sleep apnea [8]. When SP occurs in the absence of other sleep disorders, it is termed Isolated sleep paralysis (ISP)[7]. As SP is commonly associated with other sleep-altering disorders (narcolepsy, post-traumatic stress disorder, anxiety disorders, etc.), the management, when indicated, focuses on improving sleep hygiene and reducing insomnia symptoms [7]. Pharmacological treatment with tricyclic antidepressants and selective serotonin reuptake inhibitors can also be prescribed in the context of narcolepsy [7]. Additionally, several behavioral interventions, such as cognitive behavioral therapy and other psychotherapeutic approaches, have been proposed; however, reliable data is lacking [1,7].

The epidemiology of SP remains controversial. Thus, the prevalence of SP varies widely between the published individual research studies ranging from 2% to 60% [1]. This is likely due to the heterogeneities in terms of the clinical picture of SP and/or the specific diagnostic tools, whether self-questionnaires or clinical interviews [7]. Therefore, there is a need for a robust examination of the current data to be precise about how common SP is. This systematic review and meta-analysis aimed to estimate the global prevalence of SP and ISP among different populations.

## Review

Study design

A systematic review and meta-analysis were conducted to investigate the prevalence of sleep paralysis based on existing epidemiological studies. This systematic review was conducted based on the recent Preferred Reporting Items for Systematic Reviews and Meta-Analyses (PRISMA) guidelines [9]. 

Search strategy 

A comprehensive literature search was conducted to identify papers that examined the prevalence rates of sleep paralysis. The search was last performed on March 20, 2023. The search included a combination of specific terms such as "sleep paralysis," "isolated sleep paralysis," "parasomnia not otherwise specified," "hypnagogic," "hypnopompic," "parasomnia," "sensed presence," and "incubus." Additionally, the search was broadened to include indirect references to sleep paralysis, such as "sleep paralysis experiences," "nighttime paralysis," and "sleep disturbance with muscle immobility." Backward searches were also conducted to identify relevant articles to ensure thoroughness.

Data sources and screening

The search was conducted through four databases (PubMed, Scopus, Web of Science, and Cochrane Library), from inception to March 2023. Eight independent authors reviewed the included studies carefully and independently through the Rayyan online platform (Rayyan Systems, Inc., Cambridge, Massachusetts, United States). We included all populations, and there was no exclusion based on age, sex, education, residency, or mental health state. As regards the design of the included studies, we included observational studies such as cross-sectional, cohort, and case-control studies. We included studies that assessed SP as a primary or secondary outcome. Sleep paralysis is defined as the state of combined consciousness and unconsciousness in which there is a state of generalized atonia in the voluntary muscle in the REM stage. SP can occur with other psychiatric or sleep disorders or can occur in the absence of any other sleep disorder, which is termed ISP. Symptoms associated with SP and ISP are visual and auditory hallucinations. It may be combined with stress and depression and may be combined with other sleep disorders such as hypersomnia and cataplexy. Only studies in English were considered. On the other hand, we excluded reviews, conference abstracts, and non-English language studies with no English version available. There were no other restrictions made during screening. 

Data extraction

Ten authors were divided into two groups; each group extracted all the data independently, and the data was revised and compared with each other. The paper's data concerned population demographics like age, sex, and education. The prevalence of SP was extracted as a percentage from each study, and the prevalence of the total sample was extracted and calculated from studies with more than one participant group. Other data extracted was the name of a questionnaire used in each study to investigate SP, risk factors, and special habits, and data about the mental health of included participants was also extracted. 

Quality assessment

Since all the included studies were observational studies like cohorts, cross-sectional, and case controls, we used the modified version of the Newcastle-Ottawa Scale (NOS) to review the quality of the included studies [9]. Four authors reviewed the quality of the included studies, and a fifth independent author re-reviewed the quality independently. Any differences or conflicts regarding the data were discussed between all the authors during online meetings. 

Statistical analysis

We performed our analysis using the R (v.4.3.0) programming language and the “meta” package of R Studio software (Posit, Boston, Massachusetts, United States) for Windows [1]. We conducted a random-effects meta-analysis using the metaprop function, which transforms the number of patients with sleeping disorders and the total sample size in each included study into a pooled meta-analysis of proportions. Heterogeneity among the included studies was assessed by the chi-square P value and the I2 test. High heterogeneity was determined by a chi-square P value of less than 0.1 and I2 values of ≥ 50%. We performed a subgroup analysis to estimate the prevalence of sleeping disorders in each country separately. Also, we performed subgroup analysis to estimate the prevalence among different populations, such as students, psychiatric patients, and the general population. Moreover, another subgroup analysis was performed based on a sleeping paralysis-detecting questionnaire and the patients’ special habitat. Finally, we estimated the prevalence of ISP and non-ISP separately. The pooled results and weight of studies in the meta-analysis are represented in the forest plots. The probability of publication bias is represented in funnel plots and the meta-regression of variables associated with SP is represented in scatter plots. 

Meta-regression

We performed a meta-regression to explore whether there was an association between the prevalence of sleeping paralysis and female sex, sample size, or year of publication.

Protocol registration

The protocol of this systematic review and meta-analysis is registered on the International Prospective Register of Systematic Reviews (PROSPERO) with a unique registration ID: CRD42023494867.

Results

Literature Search Results

**Figure 1:**
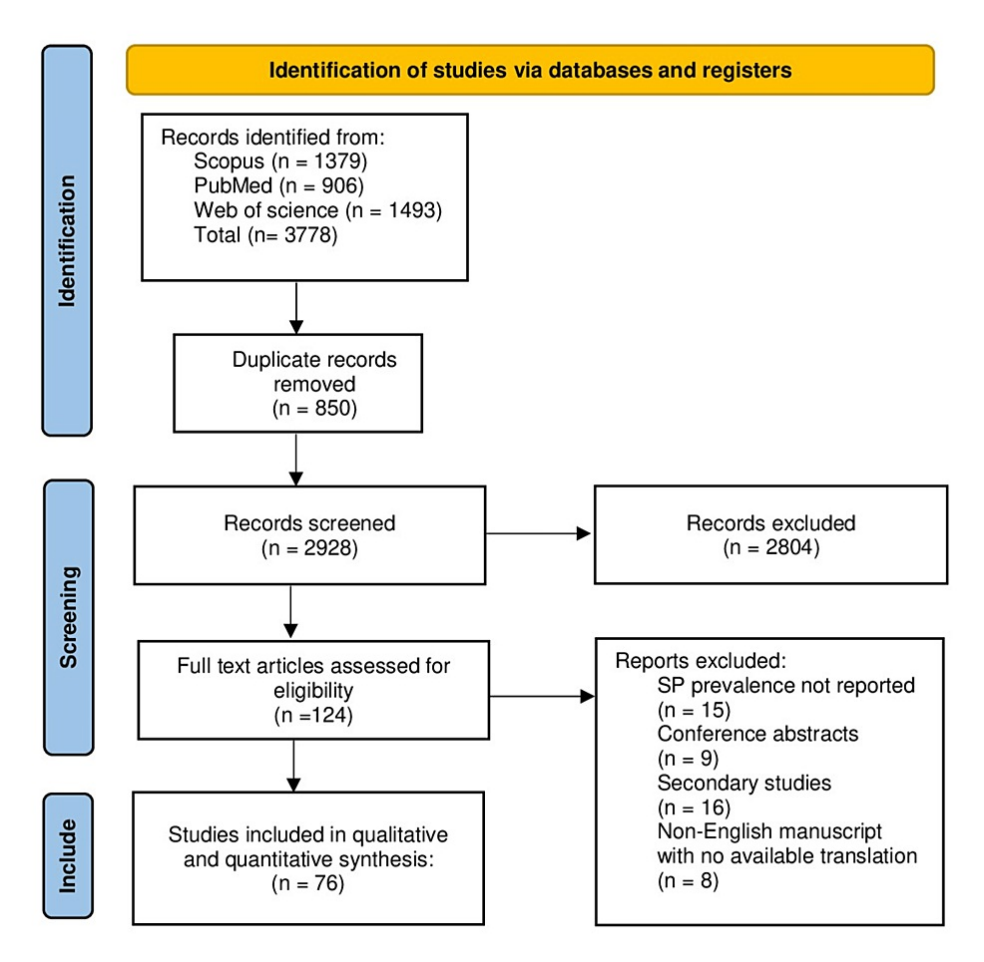
PRISMA Flow Diagram PRISMA: Preferred Reporting Items for Systematic Reviews and Meta-Analyses

Our search revealed 906 studies from PubMed, 1379 from Scopus, and 1493 from Web of Science. A total of 3778 studies have been identified through our stratified screening, as shown in the PRISMA flow diagram (Figure 1). From the identified studies, only 76 studies were included in the final qualitative and quantitative synthesis.

Characteristics of Individual Studies

Our analysis included 76 studies, compromising 167113 participants, including psychiatric patients, general populations, and others. All studies were observational conducted in 25 different countries: USA (n=15), Canada (n=6), China (n=5), Japan (n=5), Poland (n=5), France (n=4), UK (n=3), Ireland (n=2), Germany (n=2), Pakistan (n=2), Turkey (n=2), Nigeria (n=2), Czech Republic (n=2), Italy (n=1), Austria (n=1), Finland (n=1), Taiwan (n=1), Netherlands (n=1), KSA (n=1), Russia (n=1), Norway (n=1), Singapore (n=1), Spain (n=1), Paraguay (n=1), and Colombia (n=1). In addition, three studies were international, and another three were conducted in two countries. Among these 76 studies, 68 used questionnaires to detect the frequency of sleeping paralysis. Self-made questionnaires were the most popular tool used to detect sleeping paralysis, employed in 13 studies. The Waterloo Unusual Sleep Experience Questionnaire (WUSEQ) was used in five studies, the Unusual Sleep Experiences Questionnaire (USEQ) in three studies, and the Sleep Paralysis Experience and Phenomenology Questionnaire (SP-EPQ) in two studies. The remaining studies used a combination of these questionnaires and other widely used questionnaires, such as the Sleep Disorders Questionnaire (SDQ), Sleep Experiences Questionnaire (SEQ), Sleep paralysis questionnaire (SPQ), and Epworth Sleepiness Scale (ESS). According to the NOS, the majority of the included studies varied between fair and good quality, with the majority of studies being of fair quality (Tables 4, 5, 6 in the Appendices). We summarized the included studies and their patients’ characteristics (Table 1 and Table 2).

**Table 1:** Summary of the included studies MS: Multiple sclerosis; MSLT: Multiple Sleep Latency Test; OSA: Obstructive sleep apnea; GO: Ghost oppression phenomenon; SP: Sleep paralysis; ISP: Isolated sleep paralysis; PTSD: Post-traumatic stress disorder; REM: Rapid eye movement; GABA: Gamma-aminobutyric acid; NOS: Newcastle-Ottawa Scale; WUSEQ: Waterloo Unusual Sleep Experience Questionnaire; USEQ: Unusual Sleep Experiences Questionnaire; SP-EPQ: Sleep Paralysis Experience and Phenomenology Questionnaire; SDQ: Sleep Disorders Questionnaire; SEQ: Sleep Experiences Questionnaire; SPQ: Sleep Paralysis Questionnaire; ESS: Epworth Sleepiness Scale; ADHD: Attention deficit hyperactivity disorder; PSG: polysomnogram; CSF: cerebrospinal fluid; DM1: Diabetes mellitus type 1; SSI: Short Sleep Index; HH: Hypnogogic hallucinations.

Author	Year	Title	Type of study	Measures of sleep paralysis	Conclusion	Quality
Vela-Bueno et al. [10]	1999	Prevalence of sleep disorders in Madrid, Spain	Cross-sectional	Study in Madrid (1990): 1,500 adults, diverse data, disorder prevalence. Self-made modified SP questionnaires were used.	High sleep disorder prevalence in Madrid adults, similar to worldwide trends.	Fair
Poirier et al. [11]	1986	Clinical and sleep laboratory study of narcoleptic symptoms in multiple sclerosis	Cross-sectional	Studied 70 white MS patients and assessed narcoleptic symptoms via interview. A self-made SP questionnaire was used.	MS patients show high narcoleptic symptoms.	Good
Bell et al. [12]	1984	Prevalence of isolated sleep paralysis in black subjects	Cross-sectional	Surveyed 108 black subjects on isolated sleep paralysis experiences. A self-made SP questionnaire was used.	In the survey, 44 individuals reported ISP episodes.	Good
Magali et al. [13]	2020	Association between sleep quality and sleep paralysis in medical students from a private university in Paraguay	Cross-sectional	Paraguay medical students (2018): SP analysis. Prevalence ratios via regression.	Poor sleep, frequent paralysis, positive sleep quality link. Sex, age impact.	Fair
Drinkwater et al. [14]	2020	Lucid dreaming, nightmares, and sleep paralysis: associations with reality testing deficits and paranormal experience/belief	Cross-sectional	United Kingdom study: Explored lucid dreaming, nightmares, SP, and reality.	Study shows self-generated processes' role in lucid dreaming control.	Fair
Cornejo-Sanchez et al. [15]	2019	Sleepwalking and sleep paralysis: prevalence in Colombian families with genetic generalized epilepsy	Cross-sectional	Interviewed 67 cases, kin; identified epilepsy subtypes, analyzed using tests.	Fear is linked to hallucinoid experiences, notably sensed presence. Regression supports the association hypothesis.	Poor
Lacaux et al. [16]	2019	Increased creative thinking in narcolepsy	Cohort	Studied SP in 185 narcolepsy creativity in 126 controls. The Test of Creative Profile and the Creativity Achievement Questionnaires were used.	Narcolepsy symptoms are linked to higher creativity scores, excluding cataplexy.	Good
Sharpless et al. [17]	2019	Clinical features of isolated sleep paralysis	Cross-sectional	Sum: 185 ISP individuals, 322 controls assessed for symptoms, hallucinations, insomnia.	ISP episodes are complex and multisensorial, often with fear. Vivid hallucinations were common.	Good
Otsuka et al. [18]	2017	Nightmares and sleep paralysis among the general Japanese population: a nationwide representative survey	Cross-sectional	Cross-sectional survey in Japanese schools; anonymous questionnaires for all.	Reveals nightmares, sleep paralysis in Japanese adolescents. Emphasizes preventive education.	Good
Jiménez-Genchi et al. [19]	2017	Crude and adjusted prevalence of sleep complaints in Mexico City	Cross-sectional	Surveyed 1933 adults in Mexico City for sleep symptoms. SDQ was used.	High prevalence of sleep complaints and psychosocial and health issues.	Good
Schlüter et al. [20]	2016	Increased frequency of narcolepsy in childhood and adolescence: case series of a pediatric sleep laboratory (1995-2015)	Cross-sectional	In Datteln, Germany, (1995–2015): 64 narcolepsy patients, increasing over the years.	In Narcolepsy patients: Hypersomnia, cataplexy, sleep paralysis; family history noted.	Good
Vernet and Arnuf [21]	2009	Narcolepsy with long sleep time: a specific entity?	Cohort	Two groups were interviewed in a prospective cohort designed to identify narcolepsy-related disorders	Longer sleep time is associated with 18% of narcoleptics.	Fair
Sharpless et al. [22]	2017	Isolated sleep paralysis: fear, prevention, and disruption	Cross-sectional	156 undergraduates were assessed for lifetime ISP via clinical interview. The ISP questionnaire was self-made.	Disruption was a common and effective technique that encouraged usage and monitoring.	Fair
Gangdev et al. [23]	2015	Isolated sleep paralysis and hypnic hallucinations in schizophrenia	Cross-sectional	Patients with schizophrenia screened for SP issues	Studying SP and HH in schizophrenia patients is challenging; refined studies are needed.	Fair
Jalal and HInton [24]	2015	Sleep paralysis among Egyptian college students: association with anxiety symptoms (PTSD, trait anxiety, pathological worry)	Cross-sectional	100 participants in Cairo were explored for SP's relationship with anxiety symptoms. SPQ questionnaire was used.	High SP rates; more in women. Linked to PTSD, anxiety, and hallucinations.	Good
Jalal and HInton [25]	2013	Rates and characteristics of sleep paralysis in the general population of Denmark and Egypt	Cross-sectional	Compared SP rates in Denmark and Egypt. SP questionnaire was self-made.	High SP rates among Egyptians frequency, prolonged immobility, and fear of dying. Belief in the supernatural is linked to fear and longer immobility.	Fair
Cheyne et al. [26]	1999	Relations among hypnagogic and hypnopompic experiences associated with sleep paralysis	Cross-sectional	Waterloo Scale assesses sleep paralysis prevalence-related hallucinosis experiences. SP was assessed by WUSEQ.	Fear is linked to hallucinoid experiences, notably sensed presence. Regression supports the association hypothesis.	Fair
Wing et al. [27]	1999	Sleep paralysis in the elderly	Cross-sectional	Hong Kong study on mental disorders in the elderly (above 70). Employed revised GO questionnaire to assess SP's prevalence. The ghost oppression phenomenon questionnaire was used.	One-third of cases: late onset. GO+ is linked to frequent sleep disturbances. Family history in 10%.	Good
Wing et al. [28]	1994	Sleep paralysis in Chinese - ghost oppression phenomenon in Hong Kong	Cross-sectional	The responses of 603 undergraduate students to a questionnaire were analyzed. A self-made questionnaire was used.	37% experienced ghost oppression attacks, no sex difference.	Good
Paradis et al. [29]	2009	The assessment of the phenomenology of sleep paralysis: the Unusual Sleep Experiences Questionnaire (USEQ)	Cross-sectional	Pilot study: 208 college students were assessed using USEQ.	USEQ is well understood. A quarter reported SP. Linked to anxiety panic attacks.	Good
Cheyne et al. [30]	2002	Situational factors affecting sleep paralysis and associated hallucinations: position and timing effects	Cross-sectional	Sleep paralysis study: 6730 subjects, exploring body position and timing. WUSEQ was used.	SP timing impacts hallucination nature; body position has a modest effect.	Fair
Fukuda et al. [31]	1987	High prevalence of isolated sleep paralysis - kanashibari phenomenon in JAPAN	Cross-sectional	The phenomenon was studied via a questionnaire with 635 college students. A self-made questionnaire was used.	Peak age of first attack earlier in women; influenced by exogenous load and endogenous development.	Fair
Denis and Poerio [32]	2016	Terror and bliss? Commonalities and distinctions between sleep paralysis, lucid dreaming, and their associations with waking life experiences	Cross-sectional	Study tests claim that sleep paralysis and lucid dreaming relate. WUSEQ was used.	Wakefulness influences REM experiences; sleep quality affects paralysis and dreaming.	Fair
Ma et al. [33]	2014	Sleep paralysis in Chinese adolescents: a representative survey	Cross-sectional	Cross-sectional study: questionnaires given to junior and senior high students. A self-made questionnaire was used.	Female sex, alcohol, low sleep quality, and rural residence increase SP risk.	Good
Fukuda et al. [34]	1998	Recognition of sleep paralysis among normal adults in Canada and Japan	Cross-sectional	Questionnaire on sleep paralysis and narcolepsy features administered to Canadian and Japanese university students. A self-made questionnaire was used.	Similar SP prevalence in Canada and Japan, but cultural perception varies.	Good
D'Anselmo et al. [35]	2020	Creativity in narcolepsy type I: the role of dissociated REM sleep manifestations	Cross-sectional	Type 1 narcolepsy patients (66) were assessed for creative achievement, beliefs, and performance. ESS was used to assess SP.	Hypnagogic hallucinations trigger mind wandering, enhancing creative success in narcolepsy.	Poor
Denis et al. [36]	2015	A twin and molecular genetics study of sleep paralysis and associated factors	Cross-sectional	The first study on SP's heritability in twins explores genetic associations. A self-made questionnaire was used to assess SP.	Sleep paralysis is moderately heritable in young adults; explores circadian polymorphisms.	Fair
Young et al. [37]	2012	Unique sleep disorders profile of a population-based sample of 747 Hmong immigrants in Wisconsin	Cross-sectional	Investigated sleep issues in 747 Hmong immigrants compared with Wisconsin Sleep Cohort. SP was assessed by a self-made questionnaire.	Hmong profile: high sleep apnea, REM issues, cultural stressors impact.	Poor
Munezawa et al. [38]	2009	Epidemiological study of nightmare and sleep paralysis among Japanese adolescents	Cross-sectional	A survey was sent to 1164 senior high students on nightmares and sleep paralysis. PSQI was used to assess SP.	Study suggests nightmares and SP common in adolescent adults are linked to mental health and insomnia.	Good
Jimenez-Genchi et al. [39]	2009	"A dead body climbed on top of me": A study of sleep paralysis in Mexican adolescents	Cross-sectional	An online survey was conducted with 322 students in Mexico City using the Epworth Sleepiness Scale. A self-made questionnaire was used.	Mexican adolescents experience the "dead body climbed on top" phenomenon more. Sleep paralysis is linked to hallucinatory experiences in adolescence.	Good
Mellman et al. [40]	2008	Sleep paralysis, psychiatric symptoms and disorders in an adult African American population attending primary care clinics	Cross-sectional	African American participants (142) received interviews and filled out a sleep paralysis questionnaire. A self-made questionnaire was used to assess SP.	Study: Sleep paralysis common in African Americans, linked to trauma. No specific psychiatric diagnoses were identified.	Good
Dodet et al. [41]	2015	Lucid dreaming in narcolepsy	Case-control	Lucid dreamer interview with daytime and nighttime sleep monitoring conducted. SP was assessed by interview.	Narcolepsy patients were more prone to lucid dreaming without REM impact.	Fair
Han et al. [42]	2006	Childhood narcolepsy in North China	Case-control	Children with narcolepsy syndromes in North China underwent brain CT and MRI, MSLT, and serological HLA typing. Some with snoring had additional PSG before MSLT.	MSLT and HLA testing aid in the identification of children with narcolepsy.	Good
Han et al. [43]	2011	Presentations of primary hypersomnia in Chinese children	Case-control	417 children with hypersomnia complaints were evaluated using the Stanford Sleep Inventory, HLA typing, and MSLT recordings.	Many children meet the criteria for narcolepsy with cataplexy; age affects sleep paralysis. Narcolepsy with cataplexy starts at a younger age.	Fair
Rauf et al. [44]	2022	The associations between paranormal beliefs and sleep variables	Cross-sectional	A large sample (N = 8853) completed an online survey by BBC Focus Magazine. PSQI was used to assess SP.	The study suggests a linkage between paranormal beliefs and sleep-related variables.	Good
Knybel et al. [45]	2020	Prevalence and clinical picture of sleep paralysis in a Polish student sample	Cross-sectional	An online survey with 439 Polish students assessed SP's prevalence, frequency, and contributing factors. SP-EPQ was used to assess SP.	A study finds a large number of students experience recurrent SP with fear and discomfort.	Good
O’Hanlon et al. [46]	2011	Experiences of sleep paralysis in a sample of Irish university students	Cross-sectional	An online survey of 2500 students found that 83 reported experiencing SP. WUSEQ was used to assess SP.	Student population: SP is linked to hallucinations, causing intense fear.	Fair
Zhang et al. [47]	2020	Narcolepsy with cataplexy: does age at diagnosis change the clinical picture?	Case-control	Narcolepsy patients were surveyed, interviewed, and studied for sleep comparisons. The ESS was used to assess SP.	Narcoleptic children show obesity, night eating, parasomnia, and ADHD compared to adults.	Fair
Frauscher et al. [48]	2014	A prospective questionnaire study in 100 healthy sleepers: non-bothersome forms of recognizable sleep disorders are still present	Cross-sectional	The survey involved 100 subjects with undisturbed sleep and sleep disorders. PSQI was used to assess SP.	Common findings in healthy sleepers: snoring, non-bothersome sleep-related movement disorders, parasomnias. Diagnostic criteria may need frequency or discomfort consideration.	Good
Wróbel-Knybel et al. [49].	2022	Risk factors of sleep paralysis in a population of Polish students	Cross-sectional	Surveyed questionnaire among Polish students from various universities (2598 responses). SP-EPQ was used to assess SP.	The study reveals significant student experience with ISP. Mental health, lifestyle, and somatic issues affect ISP.	Good
Wierzbicka et al. [50]	2006	Differential diagnosis of narcolepsy and other hypersomnias based on clinical features and neurophysiological assessment	Cross-sectional	Patients referred with suspected narcolepsy were studied for clinical, MSLT, and various scales.	Recommended for suspected narcolepsy: MSLT and polysomnographic tests in sleep labs.	Good
Mahendran et al. [51]	2006	Survey of sleep problems amongst Singapore children in a psychiatric setting	Cross-sectional	The study assessed sleep issues and psychiatric diagnoses in clinic children. A self-made questionnaire was used to assess SP.	The study stresses consulting child psychiatrists on common, underreported child sleep disorders.	Fair
Otto et al. [52]	2006	Rates of isolated sleep paralysis in outpatients with anxiety disorders	Cross-sectional	Outpatients with panic and social anxiety. Pre-treatment assessments were conducted. SEQ, and self-made instrument derived from the Waterloo Sleep Experiences Scale was used to assess SP.	High ISP rates in anxiety patients but no independent link to antidepressant/anxiolytic use.	Fair
Nelson et al. [53]	2006	Does the arousal system contribute to near-death experiences?	Cross-sectional	Subjects were chosen from a registry; those with acute danger episodes were interviewed based on Greyson criteria (score ≥ 7). A self-made questionnaire was used.	Subjects with near-death experience (NDE) reported significantly more REM intrusion than control subjects.	Good
Hinton et al. [54]	2005	Sleep paralysis among Cambodian refugees: association with PTSD diagnosis and severity	Cross-sectional	The interview assessed sleep paralysis, PTSD, diagnosed PTSD, and panic attacks.	Common sleep paralysis in Cambodian refugees, especially with PTSD. Clinicians assess during treatment.	Fair
Paradis et al. [55]	2005	Sleep paralysis in African Americans with panic disorder	Cross-sectional	Sequential study on anxiety patients, comparing African-American and white groups. A 38-item questionnaire adapted from Bell and colleagues was used.	African Americans, especially those with panic disorder, show increased recurrent SP.	Good
Yeung et al. [56]	2005	Prevalence and illness beliefs of sleep paralysis among Chinese psychiatric patients in China and the United States	Cross-sectional	Research on SP in Chinese psychiatric patients in Boston/Shanghai. A self-made questionnaire was used.	PTSD or panic disorder correlates with higher SP prevalence.	Good
Hinton et al. [57]	2005	‘The ghost pushes you down’: sleep paralysis-type panic attacks in a Khmer refugee population	Cross-sectional	Psychiatric clinic patients were assessed for recent SP frequency and PTSD. SP Frequency Questionnaire, SP Visual Hallucinations Questionnaire, and Panic Attack Symptom Questionnaire were used.	PTSD patients have elevated SP prevalence; 60% report monthly episodes.	Good
McNally et al. [58]	2004	Sleep paralysis in adults reporting repressed, recovered, or continuous memories of childhood sexual abuse	Cross-sectional	Newspaper ads, memory interviews, categorized subgroups, and a self-administered SP questionnaire were used.	The continuous memory group had the highest SP prevalence.	Fair
Kotorii et al. [59]	2001	Questionnaire relating to sleep paralysis	Cross-sectional	Sleep survey: general population, psychiatric staff, students. A detailed questionnaire on SP.	39.6% had prior SP experience.	Good
Ohayon et al. [60]	2000	Sleep disturbances and psychiatric disorders associated with posttraumatic stress disorder in the general population	Cross-sectional	Phone poll: 1,832 individuals, 15-90 years, Metropolitan Toronto. Sleep-EVAL system used.	Trauma survivors exhibit higher PTSD prevalence and mental health impact. Urges further research on complex links.	Good
Ohayon et al. [61]	1999	Prevalence and pathologic associations of sleep paralysis in the general population	Cross-sectional	Telephonic survey: representative sample, noninstitutionalized population, Germany and Italy. The sleep-EVAL questionnaire was used to assess SP.	The general population's SP prevalence is lower than reported. Linked with mental issues, notably five times more prevalent in anxiolytic medication users.	Fair
Hedman et al. [62]	2001	Parasomnias decline during pregnancy	Cross-sectional	Pregnant subjects underwent five questionnaires across three pregnancy stages. The Basic Nordic Sleep Questionnaire (BNSQ) was used.	Pregnancy reduces parasomnia, particularly in a primipara.	Good
Kliková et al. [63]	2021	Objective rapid eye movement sleep characteristics of recurrent isolated sleep paralysis: a case-control study	Cohort	Nineteen participants with recurrent sleep paralysis were recorded during two nights. The Beck Depression Inventory (BDI II) and the Beck Anxiety Inventory (BAI) were used.	Recurrent SP sans macrostructural features linked to high cortical activity.	Good
Hsieh et al. [64]	2010	Isolated sleep paralysis linked to impaired nocturnal sleep quality and health-related quality of life in Chinese-Taiwanese patients with obstructive sleep apnea	Cross-sectional	Sleep questionnaire and full PSG assess sleep apnea with/without paralysis. ESS was used.	No relation was established between OSA and ISP	Fair
Molendijk et al. [65]	2022	The incubus phenomenon: prevalence, frequency and risk factors in psychiatric inpatients and university undergraduates	Cross-sectional	WUSEQ is used to screen for incubus phenomena among university students and psychiatric inpatients. WUSEQ was used.	0.09 and 0.12 prevalence rates among students and psychiatry patients	Good
Knybel et al. [66]	2022	Characteristics of sleep paralysis and its association with anxiety symptoms, perceived stress, PTSD, and other variables related to lifestyle in selected high-stress exposed professions	Cross-sectional	Online survey: Polish high-stress professions, anxiety, SP relation. SP-EPQ was used to assess SP.	Confirmed anxiety-sleep paralysis link in two groups, with a higher prevalence than the general population in all.	Fair
Mayer and Fuhrmann [67]	2021	A German online survey of people who have experienced sleep paralysis	Cross-sectional	An online survey of the SP population measures frequency. USEQ was used.	Increased phenomena habituation linked to higher sleep paralysis frequency.	Fair
Riaz et al. [68]	2022	Predictors of sleep paralysis and the relationship of sleep paralysis with sleep quality in university students of Islamabad	Cross-sectional	A questionnaire was used to assess sleep quality among university students with SP. USEQ was used to assess SP.	Significance in sleep paralysis and insomnia relationship explored.	Good
Benham [69]	2020	Sleep paralysis in college students	Cross-sectional	A questionnaire intended to assess the prevalence of SP among university students and its association with stress and inadequate sleep. USEQ was used.	One-third of the students experience sleep paralysis that is directly linked to stress and lack of adequate sleep	Good
Aledili et al. [70]	2021	Perceived awareness of sleep paralysis phenomenon (old hag syndrome) and its most common risk factors among people from Al-Ahsa, Saudi Arabia	Cross-sectional	The self-reported survey analyzed SP prevalence in Al-Ahsa City, Saudi Arabia.	SP is common among Al-Ahsa citizens with widespread misbelief.	poor
Knybel et al. [71]	2021	Sleep paralysis among professional firefighters and a possible association with PTSD-online survey-based study	Cross-sectional	The questionnaire set assesses Sleep Paralysis prevalence and PTSD correlation in firefighters, considering stress and anxiety. A self-made questionnaire was used.	PTSD is strongly associated with sleep paralysis frequency and symptoms (1.86x chance). Higher in firefighters than the general population.	Fair
Jalal et al. [72]	2020	Sleep paralysis in Italy: frequency, hallucinatory experiences, and other features	Cross-sectional	Abruzzo, Italy, the general population was surveyed orally to assess SP experiences and phenomena. SP-EPQ was used.	High SP rates with hallucinations in Italy, are linked to cultural beliefs.	Fair
Jalal et al. [73]	2020	Beliefs about sleep paralysis in Turkey: Karabasan attack	Cross-sectional	Questionnaire interview assesses SP in Turkish university students, exploring cultural beliefs. SP-EPQ was used.	In Turkey, SP, known as "Karabasan," is countered by religious and supernatural practices.	Good
Ferguson et al. [74]	2021	Single center analysis of patients with H1N1 vaccine-related narcolepsy and sporadic narcolepsy presenting over the same time period	Cohort	Retrospective study on Irish narcolepsy patients, assessing age groups. A self-made questionnaire was used to assess SP.	Vaccine-related narcolepsy exceeds sporadic cases; clinically similar entities.	Good
Umm-e-Habiba et al. [75]	2021	Prevalence of sleep paralysis among hostelite females of University of Lahore, pakistan	Cross-sectional	Cross-sectional study: 106 female hostelites, self-administered questionnaire, convenient sampling.	University of Lahore hostelites show mild-moderate sleep paralysis prevalence prominent symptoms.	Good
Raduga et al. [76]	2020	Is there a relation among REM sleep dissociated phenomena, like lucid dreaming, sleep paralysis, out-of-body experiences, and false awakening?	Cross-sectional	Live Moscow survey with specific questions. A self-administered questionnaire was used to assess SP.	Cross-correlations in REM sleep phenomena (lucid dreaming, sleep paralysis, etc.) were revealed by the survey.	Fair
Mume and IKem [77]	2009	Sleep paralysis and psychopathology	Case-control	The survey assesses three-month ISP prevalence anxiety using the Hamilton Scale. Self-designed questionnaire based on the Hamilton Anxiety Rating Scale was used.	ISP occurs in healthy individuals but is more common in association.	Good
Laberge et al. [78]	2009	A polysomnographic study of daytime sleepiness in myotonic dystrophy type 1	Cohort	43 DM1 patients underwent unbiased PSG, MSLT, sleep diary, ESS, respiratory function, and narcolepsy symptoms assessment.	In DM1, subjective sleepiness is linked to cataplexy-like sleep paralysis and longer sleep. Objective sleepiness correlated with higher stage 4 sleep.	Good
Heier et al. [79]	2007	CSF hypocretin-1 levels and clinical profiles in narcolepsy and idiopathic CNS hypersomnia in Norway	Cohort	CSF hypocretin-1 was measured in narcolepsy patients (cataplexy, without), hypersomnia, and controls. A questionnaire on sleep habits, daytime sleepiness, accessory symptoms, duration, and treatment was used.	Patients with cataplexy narcolepsy have hypocretin deficiency.	Good
Oluwole [80]	2010	Lifetime prevalence and incidence of parasomnias in a population of young adult Nigerians	Cohort	Study with 276 subjects, parasomnias, alien perception, and sleep factors. A self-made questionnaire was used to assess SP.	70% of young Nigerians experienced parasomnias, influenced by sleep, alcohol, and workload.	Good
Aran et al. [81]	2010	Clinical and therapeutic aspects of childhood narcolepsy-cataplexy: a retrospective study of 51 children	Cohort	Chart review, prospective data on narcolepsy in children, treatments noted. The SSI questionnaire was used.	The study reports childhood narcolepsy features and emphasizes the safe use of adult treatments.	Good
Sharpless et al. [82]	2010	Isolated sleep paralysis and fearful isolated sleep paralysis in outpatients with panic attacks	Cross-sectional	Fearful ISP Interview assesses panic disorder patients. Hamilton's measure of anxiety and depression is a measure of the severity of panic disorder. Anxiety sensitivity index, a brief explanation questionnaire for physical sensations, was used.	29.3% met lifetime ISP criteria; 20.3% met fearful ISP criteria. Fearful ISP is associated with PTSD, body mass, and anxiety sensitivity.	Good
Munezawa et al. [83]	2010	Nightmare and sleep paralysis among Japanese adolescents: a nationwide representative survey	Cross-sectional	Cross-sectional survey in Japanese junior/senior high schools. Self-reported anonymous questionnaires were distributed to all students. A 12-item General Health Questionnaire (GHQ-12) was used.	Adolescents face nightmares and sleep paralysis; regular sleep is vital.	Good
Nevsimalova et al. [84]	2011	Clinical features of childhood narcolepsy. Can cataplexy be foretold?	Cross-sectional	Pediatric narcolepsy study: Clinical exams, PSG, MSLT, HLA-DQB1∗0602, and ESS were used.	Narcolepsy in childhood leaves very little scope for the prediction of cataplexy later in life.	Fair
Erdem et al. [85]	2012	Demographic, clinical, and polysomnographic features in patients with narcolepsy: an experience of 181 patients with narcolepsy from a Turkish sleep center	Cross-sectional	Retrospective study: 181 narcolepsy patients diagnosed (1993-2009) via clinical evaluation, PSG, and MSLT were used to assess SP.	SP is prevalent among patients with cataplexy and hallucinations. Age-related differences detected in REM sleep characteristics.	Good

**Table 2:** Baseline data of the included studies NR: Not reported; NA: Not applicable; SP: Sleep paralysis; PTSD: Post-traumatic stress disorder; CNS: Central nervous system

Author	Country	Total number of samples: N	Total number of females: N(%)	Age: mean (SD) or range	Race	Psychiatric disorders
Cheyne, et al. [26]	Canada	870	488(56%)	With SP 19.91 (2.60)/without 19.62 (2.56)	Canadian	None
Wing, et al. [27]	China	158	92(58%)	80.3 (6.53)	Chinese	NR
Wing, et al. [28]	China	603	252(42%)	20.6 (1.2)	Chinese	NR
Paradis, et al. [29]	United States	208	169(83%)	22 (NR)	American, African, Asian	NR
Cheyne, et al. [30]	International	1446	795(55%)	19.87 (2.54)	Canadian, Japanese	None
Fukuda, et al. [31]	Japan	635	245(38.58%)	19.6 (NR)	Japanese	None
Denis and Poerio [32]	International	1928	1022(53%)	34. 17 ( 13.6)	European	None
Ma, et al. [33]	China	11754	5786(49.2%)	NR	Chinese	NR
Fukuda, et al. [34]	International (Japan)	149 (Japan)	61(40.9%)	19.1 (NR)	Japanese	None
	International (Canada)	86 (Canada)	63(73.25%)	20.5 (NR)	Canadian	None
Anselmo, et al. [35]	Italy	66	31(47%)	38.62 (17.05)	European	Hypnoenic hallucination
Denis, et al. [36]	United Kingdom	862	569(66%)	25.3 (1.81)	European	None
Munezawa, et al. [38]	Japan	916	348(38%)	NR	Japanese	None
Jimenez-Genchi, et al. [39]	Mexico	322	215(66.8%)	15.9 (0.88)	Mexican	NR
Mellman, et al. [40]	United States	441	300(68%)	40 (13.3)	African Americans with different psychiatric disorders	
Dodet, et al. [41]	France	159	63(40%)	37 (14)	European	NR
Han, et al. [42]	China	29	8(27.5%)	10.7 (3.0)	Chinese	NR
Han, et al. [43]	China	417	NR	8.3 (0.16)	Chinese	Primary hypersomnia
Rauf, et al. [44]	United Kingdom	8853	5868(67%)	47.04 (15.63)	White, Mixed, Asian/Asian British, Black/Black British	Paranormal beliefs/ parasomnia
Knybel, et al. [45]	Poland	439	328(75%)	22 (3.92)	Polish	None
O’Hanlon, et al. [46]	Ireland	418	292(69.85%)	21.2 (4.6)	Irish	None
Zhang, et al. [47]	France	92	36(39%)	Children: 12 (3); adults: 28.5 (13.25)	French	Cataplexy
Frauscher, et al. [48]	Austria	100	60(60%)	43 (9.66)		None
Knybel, et al. [49]	Poland	2553	2038(79.82%)	22 (2.38)	Polish	NR
Hedman, et al. [62]	Finland	325	325(100%)	29.1 (5.2)		Parasomnia
Kliková, et al. [63]	Czech Republic	38	17(89.47%)	24.89 (6.54)		None
Hsieh, et al. [64]	Taiwan	107	20(18.7%)	51.6 (13.9)	Asian	None
Molendijk, et al. [65]	Netherlands	749	588(78.5%)	Inpatient population: 47 (14); student population: 22 (4)	Western European background	Patients with different psychiatric disorders
Knybel, et al. [66]	Poland	844	601(71.20%)	Range: 18 to 67		PTSD and anxiety
Mayer and Fuhrmann [67]	Germany	380	168(44.21%)	20.4 (10.8)		None
Riaz, et al. [68]	Pakistan	440	207(47%)	Range: 20 to 23		None
Benham [69]	United States	1115	781(70%)	20,4 (3,8)	Hispanic	None
Aledili, et al. [70]	Kingdom of Saudi Arabia	524	379 (72.3%)	21.6 ( 11.8)		None
Knybel, et al. [71]	Poland	831	27(3.35%)	Range: 18 to 51	Polish	PTSD
Jalal, et al. (1) [72]	Italy and Turkey	67	33(49%)	41.2 (17.9)		None
Jalal, et al. (2) [73]	Turkey	59	44(75%)	23,2 (2,9)	Turkish	None
Ferguson, et al. [74]	Ireland	54	27 (50%)	13.5 (6)	Irish, Black African, and White European.	None
Habiba, et al. [75]	Pakistan	106	106(100%)	Range: 18 to 26		NR
Raduga, et al. [76]	Russia	974	528(54%)	29 (15)	German	NR
Mume and IKem [77]	Nigeria	116	47 (40.5%)	Orthopedic patients: 38.1 (17.6); patients with multiple somatic complaints: 36.1 (8.5); healthy subjects (control): 37.4 (9.3)	African	None
Vernet, et al. [21]	France	900	61(6.7%)	Narcolepsy with long sleep time (>11h): 25.7 (10.6); narcolepsy without long sleep time: 30.8 (10.1); idiopathic hypersomnia with long sleep time: 29.6 (12.5); controls: 29.4 (8.7)	French	Hypersomnia
Laberge, et al. [78]	Canada	43	29(67.4%)	49.7 (10.0)	N/A	Hypersomnia
Heier, et al. [79]	Norway	64	27(42.1%)	Patients with narcolepsy plus cataplexy and low hypocretin: 50.34 (46.63). Patients with cataplexy and normal hypocretin: 47 (39.8). Patients with narcolepsy without cataplexy: 35 (40.41). Patients with idiopathic CNS hypersomnia: 42.67 (26.67). Controls: 45.34 (48.85)	Caucasian	Hypersomnia
Wierzbicka, et al. [50]	Poland	33	15(45.45%)	37.9 (14.6)	N/A	Hypersomnia
Mahendran, et al. [51]	Singapore	490	158(32.2%)	10.1(3.6)	Chinese, Malay, Indian, and other ethnicities	Children attending outpatient clinics with different psychiatric disorders
Otto, et al. [52]	United States	61	27(44.3%)	43(13)	Caucasian, African American, Asian or Pacific Islander, and others	Anxiety disorders
Nelson, et al. [53]	United States	110	72(65.45%)	Near-death experience group: 54.5 (9.65); controls: 54.5 (9.87)	North American	None
Hinton, et al. [54]	United States	100	58(58%)	55.3 (8.7)	Cambodian	PTSD
Paradis, et al. [55]	United States	101	NR	NR	African American, Afro-Caribbean, White	Panic disorders
Yeung, et al. [56]	China and the United States	194	120(61.8%)	Boston 55 (14); Shanghai: 37 (13)	Asian, African American, White	None
HINTON, et al. [57]	United States	100	68(68%)	49.1 (5.3)	Cambodian	Panic attacks
McNally, et al. [58]	United States	86	64(74.4%)	Repressed memory group: 45.9 (11.4); recovered memory group: 44.1 (15.6); continuous memory group: 39.7(10.2); control group: 40.5 (14.7)	N/A	PTSD
Kotorii, et al. [59]	Japan	8162	NR	NR	Asian	None
Ohayon, et al. [60]	Canada	1832	1,009(51.7%)	NR	Black, White, Hispanic, Asian, and others	PTSD
Jiménez-Genchi, et al. [19]	Mexico	1933	1062(60%)	34.8 ( 16.7)	Mexican	NR
Schlüter, et al. [20]	Germany	64	32(50%)	NR		NR
Sharpless, et al. [22]	United States	156	100(64%)	19.63 (2.79)	American	NR
Gangdev, et al. [23]	Canada	71	22(40%)	46.51 (11.14)	Canadian	Schizophrenia
Jalal, et al. [24]	Egypt	100	86(86%)	19.3 (1.1)	Egyptian	PTSD
Jalal, et al. [25]	Egypt and Denmark	693	285.7(41.22%)	Egypt: 30.5 (11.5); Denmark: 1.7 (13.4)	Egyptian and Danish	NR
Young, et al. [37]	United States	747	283.86(38%)	40 (13)	American	NR
Erdem [85]	Turkey	181	16(8.8%)	24.61 (6.83)	Turkish	NR
Nevsimalova, et al. [84]	Prague	30	18(60%)	14.0 (3.0)	Paraguayan	Cataplexy
Otuska, et al. [18]	Japan	90,081	NR	NR	Japanese	NR
Sharpless, et al. [82]	United States	133	88.9(66.9%)	38.8 (12.8)	American	Panic attacks
Aran, et al. [81]	United States	51	43(51%)	10.3 (0.5)	American	Cataplexy
Oluwole [80]	Nigeria	276	115(41.6%)	25 (3)	Nigerian	Parasomnias

Frequency of Sleeping Paralysis Among Different Populations

**Figure 2:**
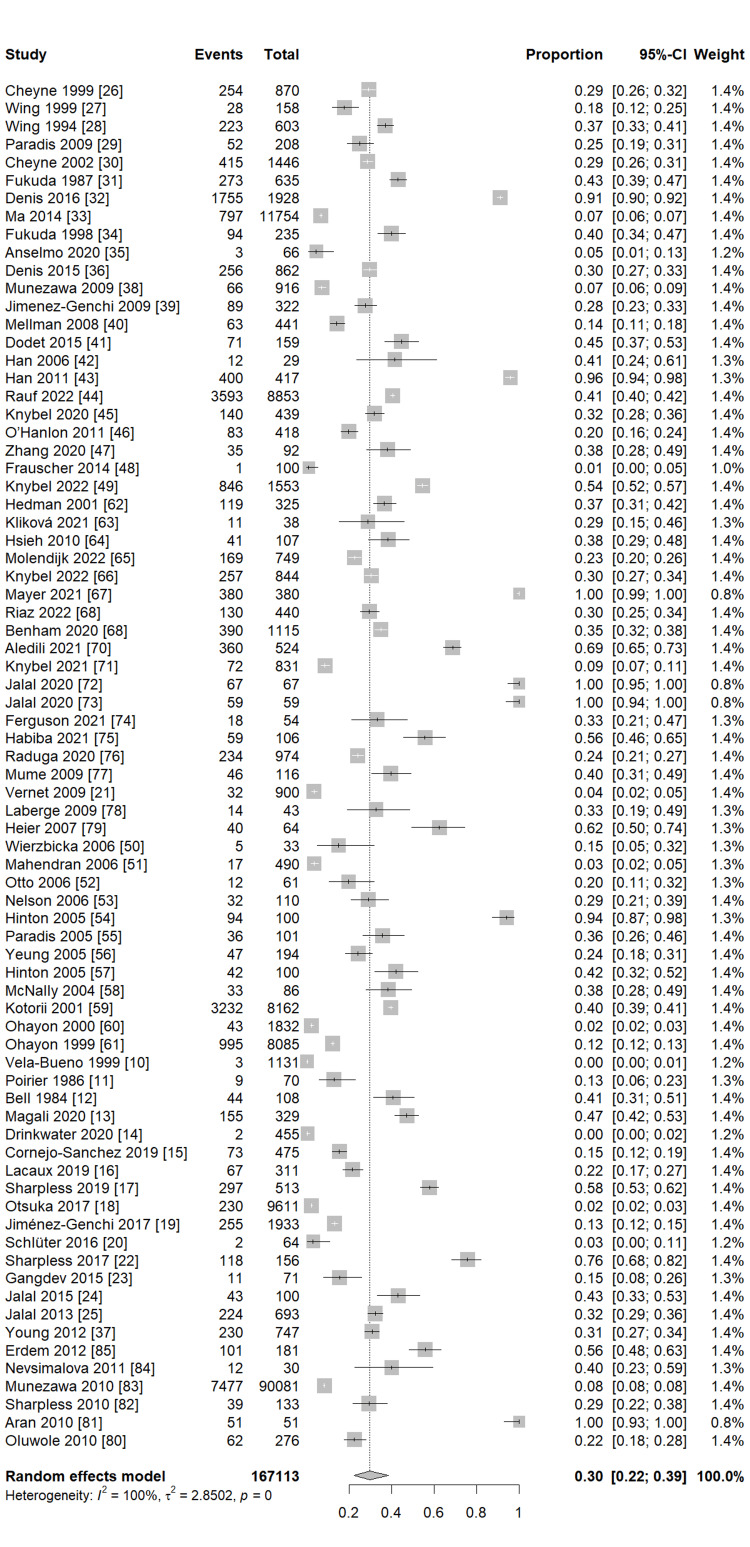
The overall prevalence of included studies

**Figure 3:**
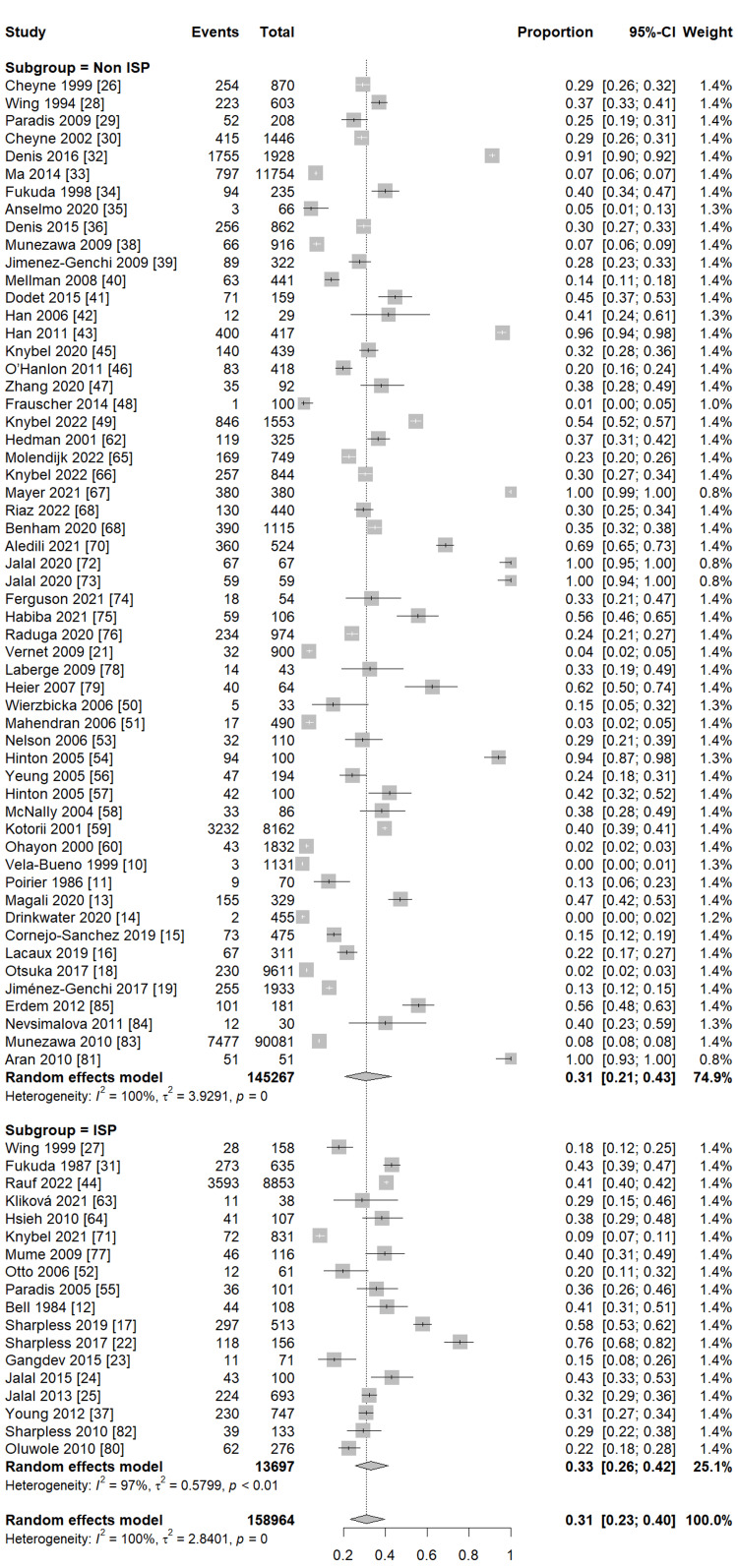
Subgroup analysis of SP and ISP SP: sleep paralysis; ISP: isolated sleep paralysis

**Figure 4:**
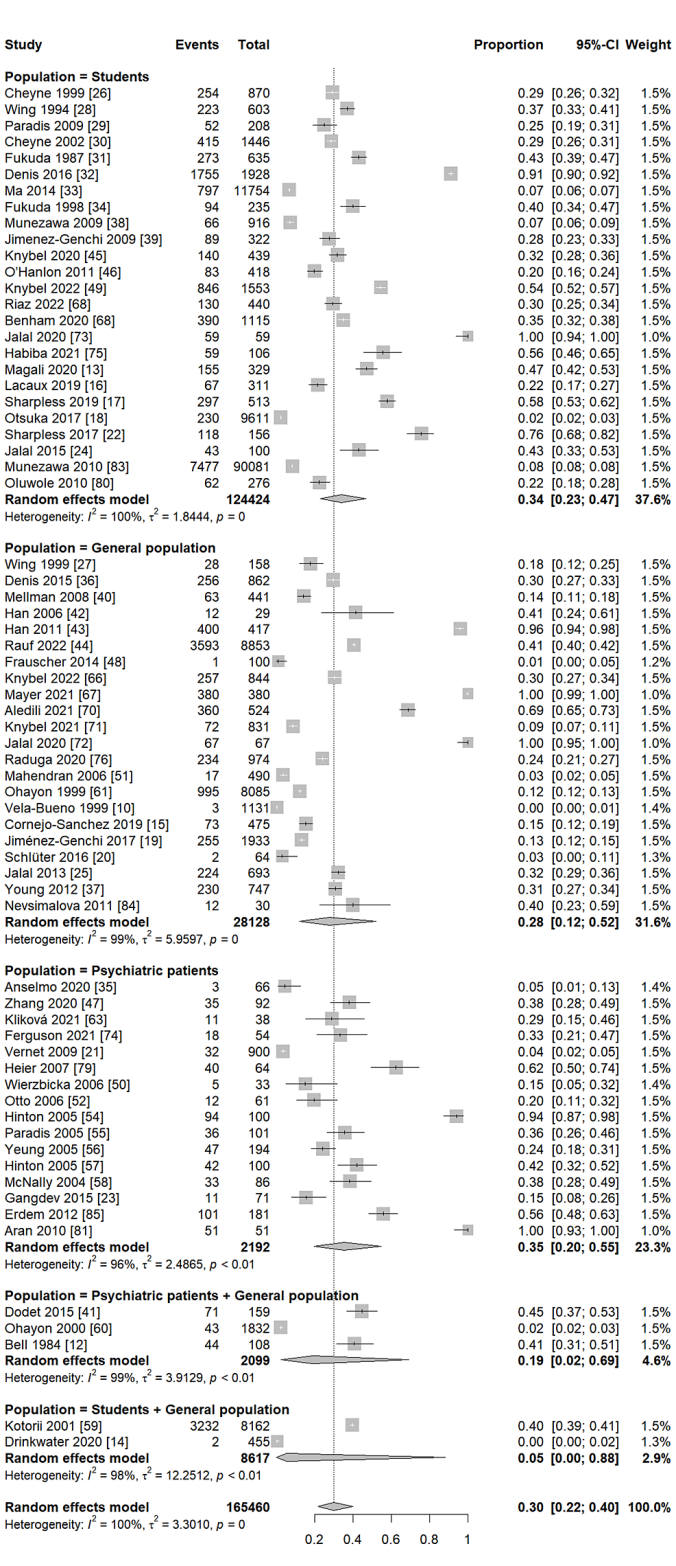
Subgroup analysis based on the prevalence of SP among populations SP: sleep paralysis

**Figure 5:**
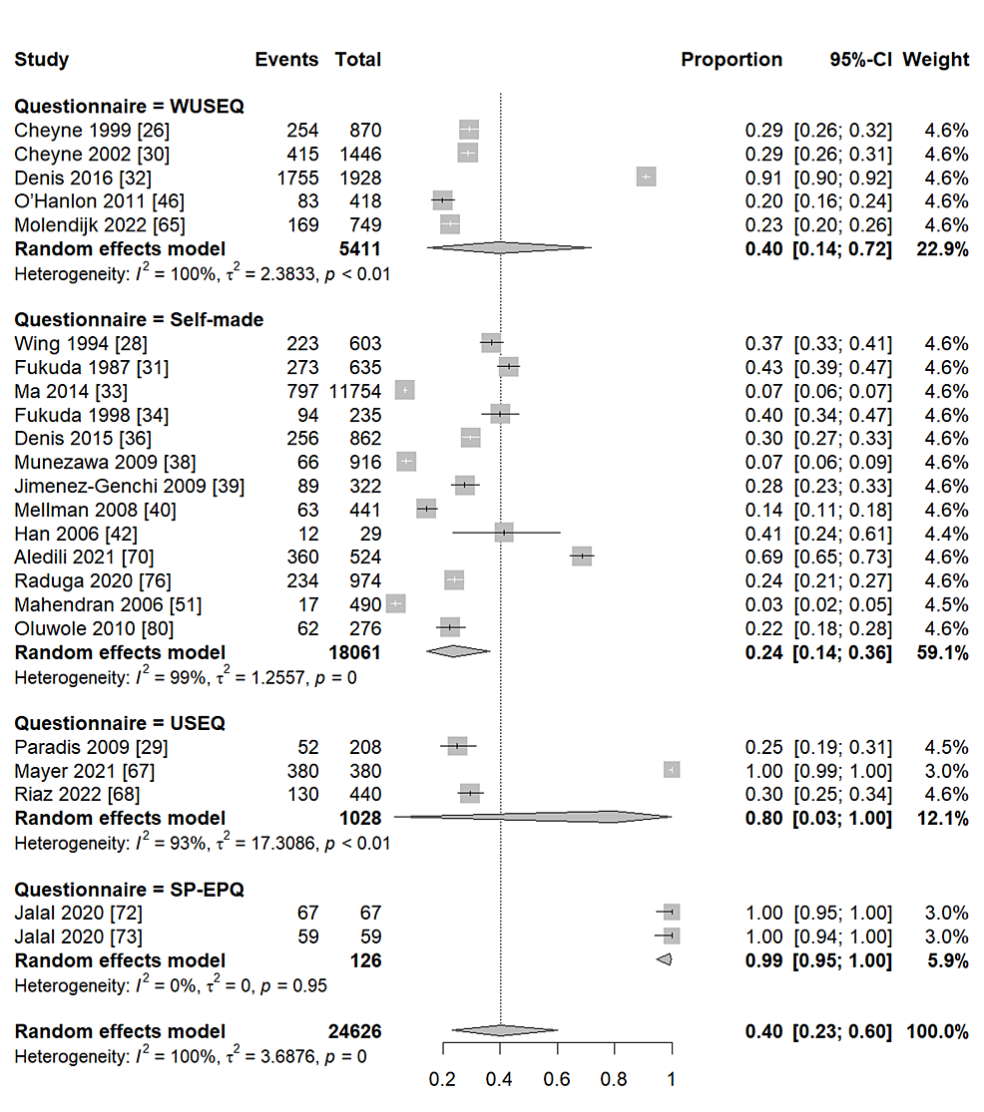
Prevalence of SP according to the type of questionnaire SP: sleep paralysis; WUSEQ: Waterloo Unusual Sleep Experience Questionnaire; USEQ: Unusual Sleep Experiences Questionnaire; SP-EPQ: Sleep Paralysis Experience and Phenomenology Questionnaire

Our pooled analysis for 76 studies revealed that the global frequency of sleeping paralysis was 30% (95% CI (22%, 39%)), with considerable heterogeneity between studies (I2 = 100%, P = 0) (Figure 2). Subgroup analysis observed a similar frequency of sleeping paralysis in studies reporting ISP and SP (33%, 95% CI (26%, 42%), I2 = 97%, P <0.01; 31%, 95% CI (21%, 43%), I2 = 100%, P = 0, respectively) (Figure 3). Furthermore, another subgroup analysis based on the different populations revealed that the highest frequency of sleeping paralysis was among psychiatric patients (35%, 95% CI (20%, 55%), I2 = 96%, P <0.01) followed by the non-psychiatric general population, particularly among students, (34%, 95% CI (23%, 47%), I2 = 100%, P = 0) (Figure 4). Finally, the lowest frequency of sleeping paralysis was observed in studies that used self-made questionnaires (24%, 95% CI (14%, 36%)) (Figure 5). Nearly 997 patients (3.8%) of patients had visual hallucinations alone in association with their sleeping paralysis; however, only 12 patients (0.04%) reported auditory hallucinations alone with no other hallucination. In addition, 6339 patients (24.25%) reported both visual and auditory hallucinations. The majority of patients (71.88%) had no hallucinations (Table 3).

**Table 3:** Hallucinations associated with SP SP: sleep paralysis

Visual	Auditory	Visual + auditory	No hallucinations	Total cases
997	12	6339	18792	26140
3.80%	0.04%	24.25%	71.88%	100%

Meta-Regression

**Figure 6:**
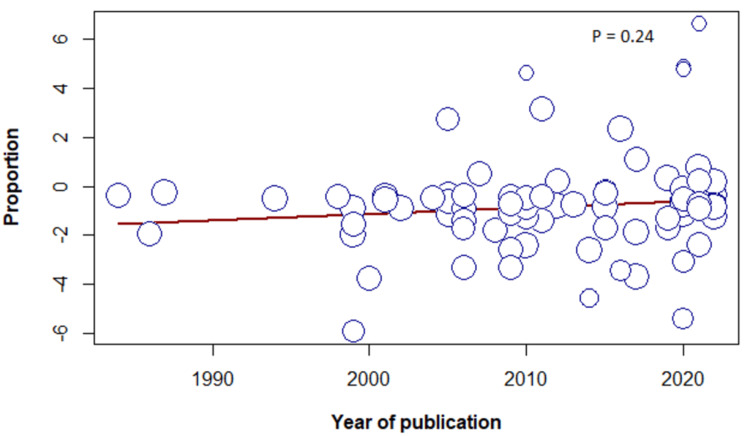
Meta-regression based on the year of publication

**Figure 7:**
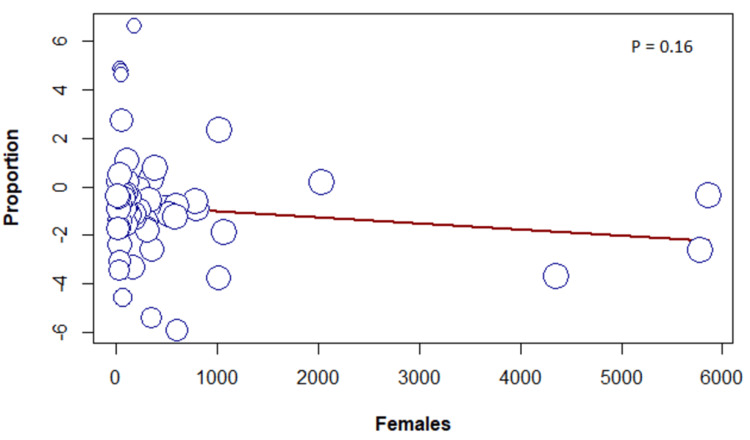
Meta-regression based on sex

**Figure 8:**
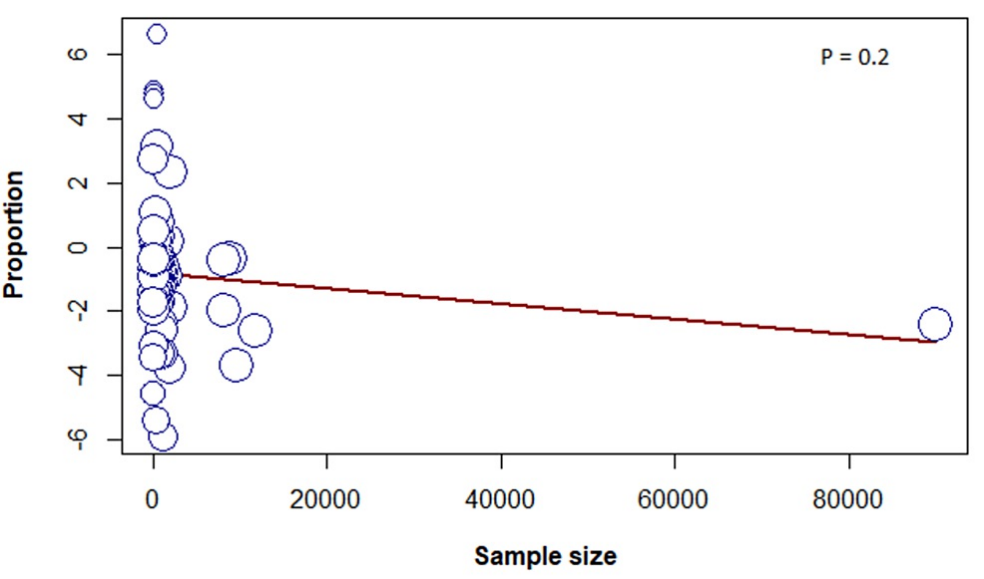
Meta-regression based on sample size

The results of the meta-regression determined no association between the frequency of sleeping paralysis and female sex, sample size, or year of publication (Figures 6-8). 

Publication Bias

**Figure 9:**
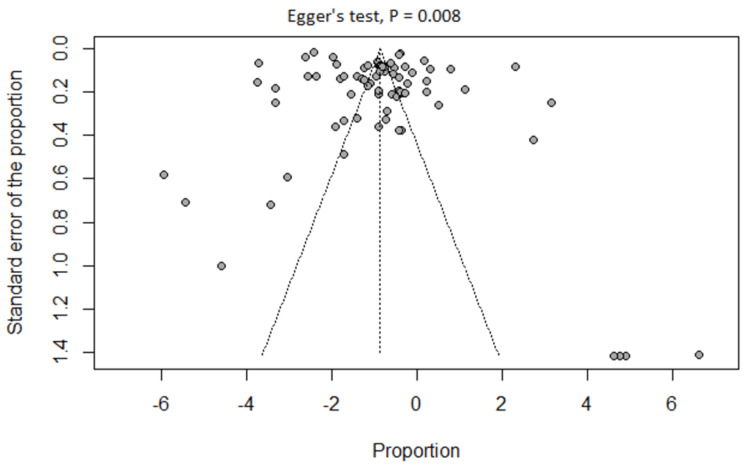
Funnel plot - Egger's test showing the probability of publication bias

**Figure 10:**
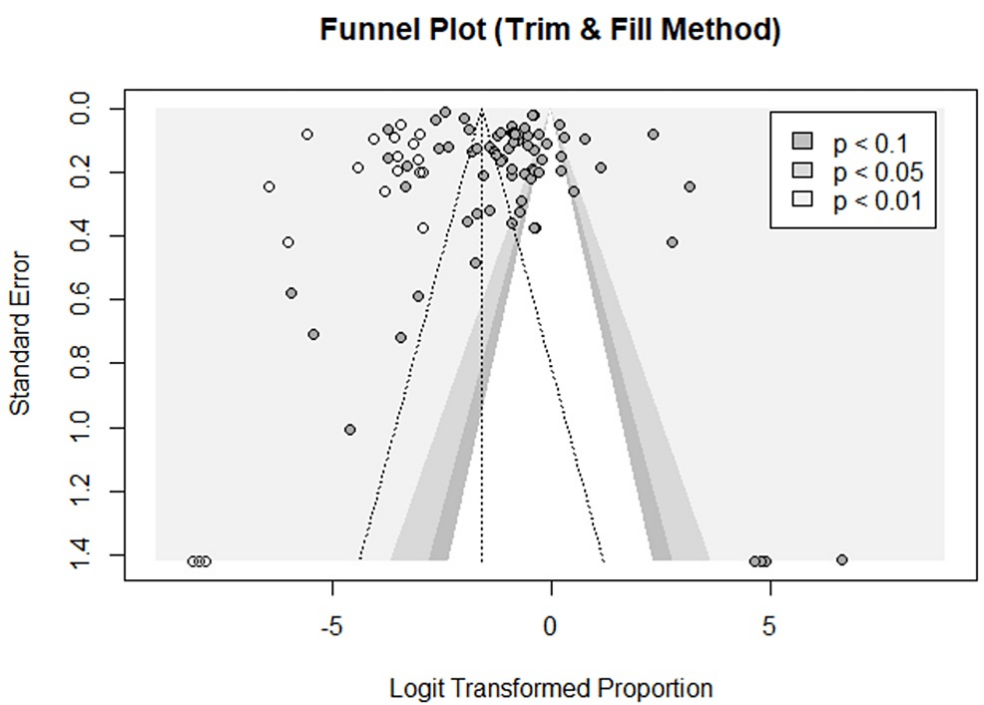
Funnel plot - the trim and fill method showing the probability of publication bias

Visual inspection of the funnel plot revealed asymmetry, which was confirmed by the significant results of Egger’s test (P = 0.008) (Figure 9). The trim and fill method showed that adding 20 studies altered the frequency of sleeping paralysis to 17% (95% CI (11%, 25%)) (Figure 10).

Discussion

Summary of the Findings

In this systematic review and meta-analysis, we found that the global prevalence of SP is estimated at 30% when data from all different populations (general population, psychiatric patients, students, and others) is combined. Notably, students and psychiatric patients were the most affected populations. Additionally, the type of questionnaire had a remarkable impact on the frequency of SP as using self-made or unvalidated questionnaires was accompanied by a significantly lower rate of SP while using validated questionnaires like the SP-EPQ, USEQ, and WUSEQ were associated with significantly higher rates. Importantly, the majority of SP patients had no associated hallucinations with SP episodes; however, more than one-fourth of SP patients experienced combined visual and auditory hallucinations. The occurrence of visual or auditory hallucination alone was unlikely. There was no difference in the prevalence of SP and ISP among the included studies.

High Prevalence of SP Among Psychiatric Patients

Our finding regarding the greater susceptibility of psychiatric patients to SP confirms the results of previous literature, such as the systematic review done by Denis et al. [86]. There is a vicious circle between psychiatric diseases and sleep disorders. Furthermore, psychiatric patients are at risk for sleep deprivation, substance abuse, and alcohol consumption; these risks are correlated positively with SP [86].

Patients with post-traumatic stress disorder (PTSD) and panic disorders display more pathological events that make them more susceptible to REM parasomnias [87]. Thus, they share the nocturnal awakenings with SP patients, which may indicate mechanistic or etiological similarities [87-88]. The features of dysregulation in REM, like sleep atonia, were observed among patients suffering from PTSD and panic disorders [87-89]. Also, it was proven that there is a positive link between autonomic system imbalance during REM and PTSD [88-90]. Furthermore, PTSD is also characterized by more fragmented REM sleep periods in the form of short duration and high frequency, which would favor the episodes of intermediate wakefulness and dreaming state seen in SP [91]. As nocturnal parasympathetic activity has a key role in controlling respiratory function during REM [92], the change in parasympathetic activities among PTSD and panic disorder patients may trigger the irregular respiration attacks that occur in the incubus state.

High Prevalence of SP Among Students

We have identified a high prevalence of SP among students, supporting the findings of Sharpless and Barber [93], who found that 28.3% of students reported experiencing at least one episode of SP during their lives. Students are particularly predisposed to SP, likely due to mental distress and sleep deprivation. For example, students with a history of SP reported greater stress levels and poorer sleep than those without [69]. In particular, at school age, there is a peak in the incidence of most of the stress-related psychiatric illnesses such as anxiety disorders and impulse-control disorders, as well as substance use disorders, which are in turn associated with an increased risk of SP [49,94]. At the age of adolescence, 60% of the individuals would have experienced at least one potentially traumatic event [95], which is also another proven promoter of SP [86]. Adding to this is the negative impact of academic burnout, which is a known mutual player with sleep disorders [96]. Besides mental fragility, students frequently have a poor healthy lifestyle that exposes them to SP, such as the greater tendency to sleep deprivation, often excessive consumption of psychoactive substances such as caffeine, and inadequate physical activity [49].

Hallucinations in SP Patients: Characteristics and Mechanism

After reviewing the available evidence, we showed that hallucinations are moderately common and are often visual and auditory during SP. Hallucinations manifest as sensorial perceptions of human or non-human entities, which are explained by patients as paranormal experiences, dreams, or strange phenomena related to supernatural powers or evil spirits [97]. The description of hallucinations is remarkably influenced by occult narratives and historical cultures [4]. Sleeping in the supine position is more associated with hallucinations occurrence [98]. These hallucinations can be considered both hypnagogic (experienced during sleep) and hypnopompic (experienced during awakening) experiences [4]. Their mechanisms are unknown, however, and are speculated to be the result of multiple neurophysiological anomalies that occur concomitantly or separately during SP episodes [5]. Thus, the phenomenon of intruder, where patients experience auditory and visual hallucinations along with a felt presence and fear sensations, is speculated to originate from a hypervigilant state initiated in the midbrain [4,5]. The unusual experiences, such as floating or flying sensations and out-of-body experiences, are thought to be caused by conflicts of intrinsic and extrinsic conscious neurological stimuli related to body position, orientation, and movement, ultimately generating physically impossible experiences [4].

The Prevalence of SP and ISP

We found no significant differences between the prevalence of SP and ISP. However, this finding was associated with considerable heterogeneity, which resulted from the very low number of studies reporting ISP. There is an overlap in the literature regarding SP and ISP definitions. According to the American Academy of Sleep Medicine, when SP occurs along with the absence of other psychiatric diseases affecting the quality of sleep, it is termed ISP. Hence, it is very important to consider the type of SP when reporting its prevalence [99]. 

Implications for Interventions

A key implication of our study is that SP is primarily a psychosocial and environmental stress-related disorder that predominates in psychologically vulnerable or traumatized individual groups, including psychiatric patients and students, with no impact of gender in the prevalence. Thus, this supports the inducible nature of SP following exposure to exogenous psychological stressors with possible endogenous factors such as genetics in some evidence of heritability [100]. It is acknowledged that REM sleep plays an important role in the processing and consolidation of emotional memory; in other words, it is more of a period for recovery from diurnal distressing or shameful events, described by some authors as overnight therapy [101]. However, exhibiting high levels of stress and anxiety was shown to alter the emotional repairing advantage of REM sleep [102], which would result in greater vulnerability to REM parasomnias, including SP. 

Although SP doesn’t tend to be a repetitive, terrifying everyday experience, recurrences are not rare. In a recent study, Muzammil et al. [96] found that 15% of medical students experienced at least one episode of SP per week. In contrast, in a cross-sectional study by Wrobel-Kynbel et al., 29% of the interviewed students reported at least one episode in the past month [103]. With these rates, screening for SP among at-risk categories seems to be a useful strategy to prevent undesirable repercussions of recurrent attacks on sleep, quality of life, academic efficacy, and even social interaction. For example, students who suffer from SP were shown to display reduced academic performance [104,105]. Furthermore, SP can affect not only the students’ quality of sleep but also the quantity of sleep as it can lead to the fear of sleeping generated by recurrent terrifying episodes [105]. Therefore, it should be managed, especially in fragile populations such as school-age individuals. There is no specific treatment for SP, and the main principle of treatment depends on improving sleep, relieving anxiety, and the management of associated psychiatric illness [105-107].

Recent literature suggests that SP may stem from an insufficiency of orexin, a neurotransmitter in the hypothalamus responsible for modulating the sleep-wakefulness cycle. Consequently, the supplementation of orexin is suggested as a potential preventive measure for SP and other narcoleptic conditions [106,107]. However, the current body of evidence lacks sufficient data to ascertain the efficacy of orexin supplementation, specifically in ISP. Further research is required to explore the potential mechanisms of SP and ISP to reach a definitive management and preventative strategy.

Limitations and recommendations

To our knowledge, this study is the largest to examine the available data from the literature regarding the global prevalence of SP. It enabled extensive evaluation of 76 studies reporting records from 26140 patients. It provided important findings that can have clinical implications. Nevertheless, we acknowledge several limitations that underpowered our work. First, estimating SP frequency was based on data collected from heterogeneous populations due to the large number of included studies. Second, there was an absence of explicit characterization of the SP episodes in terms of severity (impact on life), number (frequency per time), and type (intruder or incubus) in most of the included studies. Third, the risk of publication bias was suspected by visual inspection of the funnel plot and confirmed by Egger’s test and the trim and fill method. Hence, we recommend future observational studies to investigate the prevalence of SP in various communities, especially in areas where psychiatric diseases are common. Also, we recommend future randomized control trials regarding the effectiveness of interventions like exogenous orexin therapy for managing both SP and ISP. We recommend the usage of validated tools when collecting SP data. It is very important to differentiate between SP and ISP in terms of definitions, prevalence, and clinical picture in the future literature. 

## Conclusions

This systematic review and meta-analysis revealed a global prevalence of SP estimated at 30%. Students and psychiatric patients exhibited higher susceptibility to SP. The majority of SP cases lacked associated hallucinations, while a noteworthy proportion experienced combined visual and auditory hallucinations. The occurrence of either visual or auditory hallucination alone was infrequent. Further studies are needed to expand the current knowledge regarding the potential burdens of SP in different populations, particularly those with mentally vulnerable status.

## Appendices

**Table 4:** Supplementary table showing the NOS quality assessment of cohort studies NOS: Newcastle-Ottawa Scale

Study ID	NOS (cohort )									Final score
	Selection				Comparability	Outcome			Outcome score	
	Representativeness of the exposed cohort	Selection of the non-exposed cohort	Ascertainment of exposure	Demonstration that outcome of interest was not present at the start of the study	Comparability of cohorts on the basis of design or analysis controlled for confounders	Assessment of outcome	Was follow-up long enough for outcomes to occur?	Adequacy of follow-up of cohorts		
Lacaux, et al. 2019 [16]	*		*		**	*	*	*	7	Good
Kliková, et al. 2021 [63]	*			*		*	*	*	5	Fair
Ferguson, et al. 2021 [74]	*	*	*	*	**	*	*	*	9	Good
Laberge, et al. 2009 [78]	*		*	*	**	*	*	*	8	Good
Heier, et al. 2007 [79]	*	*	*	*	*	*	*	*	8	Good
Oluwole, 2010 [80]	*	*	*		**	*	*		7	Good
Aran, et al. 2010 [81]	*		*	*	*	*	*		6	Fair
Vernet, et al. 2009 [21]	*			*		*	*	*	5	Fair

**Table 5:** Supplementary table showing the quality assessment of case-control studies

Study ID	NOS (case-control )									
	Selection				Comparability	Outcome			Outcome score	
	Is the case definition adequate?	Representativeness of cases	Selection of Controls	Definition of controls	Comparability of cases and controls based on design or analysis controlled for confounders	Assessment of outcome	Was follow-up long enough for outcomes to occur?	Adequacy of follow-up of cohorts		
Mume and IKem 2009 [77]	*	*	*	*	*	*	*	*	8	Good
Zhang, et al. 2020 [47]	*			*	*		*	*	5	Fair
Dodet, et al. 2015 [41]	*	*			*	*	*		5	Fair
Han, et al. 2006 [42]	*	*	*		*	*	*		6	Good
Han, et al. 2011 [43]	*		*		*	*	*	*	6	Fair

**Table 6:** Supplementary table showing the NOS quality assessment of cross-sectional studies NOS: Newcastle-Ottawa Scale

Study ID	NOS (cross-sectional )								Final quality score
	Selection				Comparability	Outcome		Outcome score	
	Representativeness of the sample	Sample size	Non-respondents	Ascertainment of the exposure (risk factor)	The subjects in different outcome groups are comparable, based on the study design and analysis. Confounding factors are controlled.	Assessment of outcome	Statistical test		
Vela-Bueno, et al. 1999 [10]	*	*			**	*	*	6	Fair
Poirier, et al. 1986 [11]	*	*		*	*	*	*	6	Good
Bell, et al. 1984 [12]	*	*	*	*	*	*	*	7	Good
Magalí, et al. 2020 [13]	*	*			**	*	*	6	Fair
Drinkwater, et al. 2020 [14]	*	*			**	*	*	6	Fair
Cornejo-Sanchez, et al. 2019 [15]	*			*	*	*		4	Poor
Sharpless, et al. 2019 [17]	*	*	*	*	*	*	*	7	Good
Otsuka, et al. 2017 [18]	*	*		*	*	*	*	6	Good
Jiménez-Genchi, et al. 2017 [19]	*		*	*	*	*	*	6	Good
Schlüter, et al. 2016 [20]	*	*		*	*	*	*	6	Good
Sharpless, et al. 2017 [22]	*	*			*	*	*	5	Fair
Gangdev, et al. 2015 [23]	*		*		*	*	*	5	Fair
Jalal, et al. 2015 [24]	*	*	*	*	**	*	*	8	Good
Jalal, et al. 2013 [25]	*	*			*	*	*	5	Fair
Cheyne, et al. 1999 [26]	*	*			**	*	*	6	Fair
Wing, et al. 1999 [27]	*	*	*	*	*	*	*	7	Good
Wing, et al. 1994 [28]	*	*	*	*	*	*	*	7	Good
Paradis, et al. 2009 [29]	*	*		*	*	*	*	6	Good
Cheyne, et al. 2002 [30]	*	*			*	*	*	5	Fair
Fukuda, et al. 1987 [31]	*	*			**	*	*	6	Fair
Denis and Poerio, 2016 [32]	*	*			*	*	*	5	Fair
Ma, et al. 2014 [33]	*	*	*	*	*	*	*	7	Good
Fukuda, et al. 1998 [34]	*	*	*	*	*	*	*	7	Good
Anselmo, et al. 2020 [35]	*			*	*	*		4	Poor
Denis, et al. 2015 [36]	*	*			*	*	*	5	Fair
Young, et al. 2012 [37]	*	*	*		*	*		5	Poor
Munezawa, et al. 2009 [38]	*	*		*	*	*	*	6	Good
Jimenez-Genchi, et al. 2009 [39]	*	*	*	*	*	*	*	7	Good
Mellman, et al. 2008 [40]	*	*	*	*	*	*	*	7	Good
Rauf, et al. 2022 [44]	*	*	*	*	**	*	*	8	Good
Knybel, et al. 2020 [45]	*	*	*	*	*	*	*	7	Good
O’Hanlon, et al. 2011 [46]	*	*			**	*	*	6	Fair
Frauscher, et al. 2014 [48]	*	*	*	*	*	*	*	7	Good
Knybel, et al. 2014 [49]	*	*		*	*	*	*	6	Good
Wierzbicka, et al. 2006 [50]	*	*	*	*	*	*	*	7	Good
Mahendran, et al. 2006 [51]	*			*	**	*	*	6	Fair
Otto, et al. 2006 [52]	*	*			*	*	*	5	Fair
Nelson, et al. 2006 [53]	*	*	*	*	*	*	*	7	Good
Hinton et al. (a), 2005 [54]	*	*			*	*	*	5	Fair
Paradis, et al. 2005 [55]	*	*	*	*	*	*	*	7	Good
Yeung, et al. 2005 [56]	*	*		*	*	*	*	6	Good
Hinton, et al. (b), 2005 [57]	*	*		*	*	*	*	6	Good
McNally, et al. 2004 [58]	*	*			**	*	*	6	Fair
Kotorii, et al. 2001 [59]	*	*	*	*	*	*	*	7	Good
Ohayon, et al. 2000 [60]	*	*	*	*	**	*	*	8	Good
Ohayon, et al. 1999 [61]	*	*			**	*	*	6	Fair
Hedman, et al. 2001 [62]	*	*	*	*	*	*	*	7	Good
Hsieh, et al. 2010 [64]	*	*			*	*	*	5	Fair
Molendijk, et al. 2022 [65]	*	*	*	*	*	*	*	7	Good
Knybel, et al. (a), 2022 [66]	*	*			*	*	*	5	Fair
Mayer, and ruhmann, 2021 [67]	*	*			**	*	*	6	Fair
Riaz, et al. 2022 [68]	*	*		*	*	*	*	6	Good
Benham, 2020 [69]	*	*	*	*	*	*	*	7	Good
Aledili, et al. 2021 [70]	*		*	*	*	*		5	poor
Knybel et al. (b), 2021 [71]	*	*			*	*	*	5	Fair
Jalal, et al. 2020 [72]	*			*	*	*	*	5	Fair
Jalal, et al. 2020 [73]	*	*		*	*	*	*	6	Good
Habiba, et al. 2021 [75]	*	*	*	*	**	*	*	8	Good
Raduga, et al. 2020 [76]	*	*			*	*	*	5	Fair
Sharpless, et al. 2010 [82]	*	*		*	*	*	*	6	Good
Munezawa, et al. 2010 [83]	*	*	*	*	*	*	*	7	Good
Nevsimalova, et al. 2011 [84]	*	*			*	*	*	5	Fair
Erdem, et al. 2012 [85]	*	*		*	*	*	*	6	Good
